# Leveraging diverse cell-death patterns to predict the clinical outcome of immune checkpoint therapy in lung adenocarcinoma: Based on muti-omics analysis and vitro assay

**DOI:** 10.32604/or.2023.031134

**Published:** 2023-12-28

**Authors:** HONGYUAN LIANG, YANQIU LI, YONGGANG QU, LINGYUN ZHANG

**Affiliations:** 1 Department of Radiology, Shengjing Hospital of China Medical University, Shenyang, China; 2 Department of Nephrology, The First Affiliated Hospital of China Medical University, Shenyang, China; 3 Department of Clinical Medicine, China Medical University, Shenyang, China; 4 Department of Medical Oncology, The First Hospital of China Medical University, Shenyang, China

**Keywords:** Lung adenocarcinoma, Programmed cell death, Iron-death, Drug sensitivity, Cancer therapy

## Abstract

Advanced LUAD shows limited response to treatment including immune therapy. With the development of sequencing omics, it is urgent to combine high-throughput multi-omics data to identify new immune checkpoint therapeutic response markers. Using GSE72094 (n = 386) and GSE31210 (n = 226) gene expression profile data in the GEO database, we identified genes associated with lung adenocarcinoma (LUAD) death using tools such as “edgeR” and “maftools” and visualized the characteristics of these genes using the “circlize” R package. We constructed a prognostic model based on death-related genes and optimized the model using LASSO-Cox regression methods. By calculating the cell death index (CDI) of each individual, we divided LUAD patients into high and low CDI groups and examined the relationship between CDI and overall survival time by principal component analysis (PCA) and Kaplan-Meier analysis. We also used the “ConsensusClusterPlus” tool for unsupervised clustering of LUAD subtypes based on model genes. In addition, we collected data on the expression of immunomodulatory genes and model genes for each cohort and performed tumor microenvironment analyses. We also used the TIDE algorithm to predict immunotherapy responses in the CDI cohort. Finally, we studied the effect of PRKCD on the proliferation and migration of LUAD cells through cell culture experiments. The study utilized the TCGA-LUAD cohort (n = 493) and identified 2,901 genes that are differentially expressed in patients with LUAD. Through KEGG and GO enrichment analysis, these genes were found to be involved in a wide range of biological pathways. The study also used univariate Cox regression models and LASSO regression analyses to identify 17 candidate genes that were best associated with mortality prognostic risk scores. By comparing the overall survival (OS) outcomes of patients with different CDI values, it was found that increased CDI levels were significantly associated with lower OS rates. In addition, the study used unsupervised cluster analysis to divide 115 LUAD patients into two distinct clusters with significant differences in OS timing. Finally, a prognostic indicator called CDI was established and its feasibility as an independent prognostic indicator was evaluated by Cox proportional risk regression analysis. The immunotherapy efficacy was more sensitive in the group with high expression of programmed cell death models. Relationship between programmed cell death (PCD) signature models and drug reactivity. After evaluating the median inhibitory concentration (IC50) of various drugs in LUAD samples, statistically significant differences in IC50 values were found in cohorts with high and low CDI status. Specifically, Gefitinib and Lapatinib had higher IC50 values in the high-CDI cohort, while Olaparib, Oxaliplatin, SB216763, and Axitinib had lower values. These results suggest that individuals with high CDI levels are sensitive to tyrosine kinase inhibitors and may be resistant to conventional chemotherapy. Therefore, this study constructed a gene model that can evaluate patient immunotherapy by using programmed cell death-related genes based on muti-omics. The CDI index composed of these programmed cell death-related genes reveals the heterogeneity of lung adenocarcinoma tumors and serves as a prognostic indicator for patients.

## Introduction

Lung adenocarcinoma (LUAD) represents one of the most prevalent subtypes of non-small cell lung cancer (NSCLC) and continues to present as a recurring cause of cancer-induced mortalities globally [1]. In spite of continuous progress in the identification and treatment of LUAD, patients with late-stage disease continue to face adverse overall survival (OS) outcomes, given the limited range of available therapeutic interventions [2].

Apoptosis is a fundamentally regulated biological process that causes the death of cells. This mechanism plays a vital role in preserving tissue equilibrium, checking tumor formation, and eliminating impaired or infected cells [3]. The perturbation of programmed cell death (PCD) has been affiliated with the initiation and advancement of various cancerous conditions in humans, encompassing LUAD [4]. Aberrations in the expression and functioning of apoptosis-related genes reveal a cancer hallmark and can encourage cancer cell survival, division, and metastasis [5].

In recent times, researchers have made remarkable strides in identifying differentially expressed genes (DEGs) in LUAD and their connection with cancer progression, metastasis, and patient outcome. Nevertheless, the significance of apoptosis-related genes in LUAD prognosis, and their potential use as prognostic biomarkers, remains inconclusive. Therefore, in this research, our study aimed to identify and validate the prognostic significance of apoptosis-associated genes in LUAD by leveraging publicly available gene expression data and integrating clinical characteristics from diverse datasets.

To achieve our aim, we employed a two-step approach. The first step involved the identification of DEGs between LUAD and normal tissues. We performed this analysis using publicly available genetic data and selecting only apoptosis-related genes for our study. In the second step, we performed survival analysis employing the Cox proportional hazards model to discern genes linked with the prognostic outcome of LUAD. Subsequently, we employed least absolute shrinkage and selection operator (LASSO) regression analysis to streamline the pool of potential genes and construct a prognostic risk score model. After identifying candidate genes, we evaluated their contribution to tumor progression and patient survival using *in vitro* and *in vivo* assays. These assays included CDI, proliferation, apoptosis, and migration assays. We also performed genomic variation analysis using various bioinformatics tools, including gene set variation analysis (GSVA), to discern disparities in biological mechanisms among subpopulations categorized by gene characteristics.

Additionally, our study aims to shed light on the potential biological processes and pathways involved in LUAD pathology and identify new targets for therapeutic intervention. The development of an accurate prognostic model for LUAD could benefit patients by facilitating early detection and personalized treatment plans. It could also help clinicians identify patients who may be at high risk for poor outcomes and recommend the most appropriate treatment options. Finally, our study may contribute to the larger body of knowledge in the field of cancer genetics, assisting in the progression of our comprehension of intricate molecular pathways involved in neoplastic advancement and the concomitant mortality associated with cancer.

## Materials and Methods

### Data collection

The present investigation entailed the selection of GSE72094 [6] and GSE31210 [7] gene expression profiling data, which have not been previously examined in a comprehensive manner, from the GEO database (https://www.ncbi.nlm.nih.gov/geo/).

It is noteworthy that this research was not conducted utilizing human tissue specimens but rather utilized two sets of microarray data that were sourced from the GEO database. Therefore, in accordance with current Chinese regulations, the examination did not necessitate endorsement from an Institutional Review Board or Human Research Ethics Committee, nor did it entail the acquiescence of the participants.

### Identification of death-related genes and their association with clinical characteristics in LUAD

To discern DEGs, we applied the “edgeR” package and set the adjusted criteria of *p* < 0.05 and | log_2_FC | > 1 [8]. In addition, we employed the “maftools” computational framework [9] to investigate the concealed somatic mutation landscape in patients with LUAD. This approach allowed us to uncover previously unexplored mutational events that may have remained undetected using conventional methods. Moreover, we extracted copy number variation (CNV) measurements of PCD-associated genes and classified those exhibiting a value exceeding 0.2 as “gain”, while those with a value less than −0.2 as “loss”. Lastly, we employed the “circlize” R package [10] to visualize the different features of the death-related genes in a circular plot.

### Functional enrichment analysis

Using the “clusterProfiler” R package, utilizing the DEGs acquired from our analysis [11], we successfully discerned prospective biological pathways that may play a pivotal role in the observed phenomenon. Furthermore, we utilized GSVA to investigate the discrepancies in biological functionalities detected between the high and low CDI groups [12].

### Construction of the prognostic model based on death-related genes in LUAD

The objective of this research was to develop a prognostic model based on genes related to mortality in LUAD. To achieve this, Initially, we evaluated the impact of these genes on the survival outcome of LUAD by utilizing the univariate Cox proportional hazards regression model. Subsequently, the LASSO-Cox regression approach was employed to effectively diminish the pool of potential candidate genes and construct an optimal model that best aligns with the data. This was achieved through the selection of the “lambda.min” value using the R software package “glmnet” [13]. The model computed the CDI for every individual using the subsequent equation:

CDI=∑(βi∗Ei) 

In this context, βi signifies the hazard coefficient, while Ei denotes the gene’s expression magnitude. To enhance the clarity of the figures, we utilized a linear transformation to adjust CDI. This procedure encompassed the subtraction of the computed CDI from the minimum value, followed by its division by the maximum value, resulting in a standardized representation. This mapping of the CDI to the 0–1 range made it more accessible and easy to interpret. Based on the median CDI value, we classified individuals with LUAD into two distinct cohorts: low-CDI and high-CDI. We conducted Principal Component Analysis (PCA) using the “stats” package, and Kaplan-Meier (KM) analysis using the “survival” and “survminer” packages to examine the correlation between CDI and OS time.

### Unsupervised clustering of death-related model genes

We executed consensus clustering (CC) to discover unknown subtypes of LUAD [14] based on model genes, using the “ConsensusClusterPlus” tool. The setting for “maxK” was 10, “clusterAlg” was “km”, and “distance” was “pearson”. Based on the cumulative distribution function (CDF) curve, we selected the value of K corresponding to the smoothest and most stable curve as the number of clusters.

### Independent prognostic value of CDI

Medical Data, encompassing parameters such as patient age, tumor size (T), lymph node involvement (N), and disease stage, were gathered from the TCGA cohort of LUAD patients, and their correlation with CDI was evaluated using both univariate and multivariate Cox regression analyses. We assessed the impact of a certain factor on prognosis based on a significance level of *p* < 0.05. Factors with a Hazard Ratio (HR) greater than 1 were considered prognostic risk factors, while those with an HR less than 1 were considered prognostic protective factors.

### Establishment of nomogram

We used multivariate Cox and stepwise regression analysis to create prognostic nomograms that incorporated clinical characteristics (age, T, N, and stage) and CDI. These nomograms were displayed using the “regplot” software package. Furthermore, in our investigation, we utilized calibration graphs and conducted Decision Curve Analysis (DCA) to enhance the robustness and usefulness of our findings with the “caret” and “rmda” R packages, respectively, to assess their efficacy. The Receiver Operating Characteristic (ROC) assessment was conducted utilizing the “timeROC” R library [15] to evaluate the discriminative accuracy of the model. Furthermore, we created dynamic nomograms with the “rsconnect” and “DynNom” computer programs.

### Tumor microenvironment analysis and drug sensitivity prediction

Furthermore, our study systematically collected expression data pertaining to established immune-modulating genes and model genes for each cohort, in conjunction with the analysis of clinical characteristics and CDI. We acquired tumor microenvironment scores computed through diverse algorithms [16–18] and performed an extensive analysis to investigate the potential correlation between CDI and the expression of immunomodulators as well as the abundance of distinct immune cell populations. We obtained drug sensitivity data files from the TIDE website (http://tide.dfci.harvard.edu) and subsequently performed drug sensitivity prediction using the “oncoppredict” package [19]. Based on the aforementioned methodology, we predicted and analyzed the immunotherapy response in the CDI cohort [20].

### Cell culture

To conduct cell-based experiments, we cultured A549 and NCI-H292 cell lines in Dulbecco’s Modified Eagle’s Medium (DMEM) complete medium and Roswell Park Memorial Institute (RPMI) 1640 complete medium supplemented with 10% FBS and the specimens were placed within an incubator, set at a temperature of 37°C, and with a 5% CO_2_ concentration, to foster the required environmental conditions. The cellular samples were meticulously distributed, with an initial seeding density of 4 × 10^5^ cells per well, within a standardized 6-well culture plate, and the cell fusion status was observed after an overnight incubation for subsequent experiments. We selected cells from the logarithmic growth phase of A549 and NCI-H292 cell lines for grouping, which included a Si-NC group (control group), si1-PRKCD group, and si2-PRKCD group. The sequences of si1-PRKCD are as follows:

sense: AUACUGAACAGACAUCAACAC;

anti-sense: GUUGAUGUCUGUUCAGUAUUU.

The sequences of si2-PRKCD are as follows:

sense: UGAUGUAGUGGAUUUUGGCCU;

anti-sense: GCCAAAAUCCACUACAUCAAG.

### The impact of PRKCD on the proliferation and migration of lung adenocarcinoma cells

To initiate cell culture, tumor cells are counted using a cell counter or an automated counter and distributed into appropriate culture dishes. Typically, 1–2 mL of culture medium and 1,000–5,000 tumor cells per dish are added. The culture dishes are then placed in a suitable environment, such as a constant temperature incubator, and cultured under constant temperature conditions of 37°C and an oxygen atmosphere condition of 5% CO_2_. The morphological characteristics and proliferative behavior of neoplastic cells are assessed at various time points during the cultivation process. Upon completion of a specific cultivation period, formalin or ethanol may be employed to fix cells. Various staining agents, such as acetic acid rosin violet and Grim III stain, may be used to facilitate observation of the formation and quantity of cell clones. By utilizing Transwell chambers, A549 and NCI-H292 cell suspensions are divided into three groups: a normal cell group (si-NC), the first PRKCD gene knockdown group (si1-PRKCD), and the second PRKCD gene knockdown group (si2-PRKCD). These groups are then added to PBS and subsequently cultured in DMEM medium supplemented with 15% FBS for 24 hours. Afterward, the Transwell chambers are removed, washed with PBS, fixed with polyformaldehyde, and ultimately treated with 0.1% crystal violet dye. Five randomly chosen microscopic fields are selected and imaged to quantify the population of cells with transmembrane characteristics. The ratio of the number of transmembrane cells in the experimental group to the control group is compared to evaluate changes in cell migration ability.

### Statistical analysis

The statistical analyses were performed employing the R software (version 4.1.0). Inter-group comparisons were conducted utilizing either the student’s *t*-test or the Wilcoxon test, while the Kruskal-Wallis analysis was employed to evaluate statistical differences among multiple cohorts. Survival analysis was executed through the generation of KM survival plots. The log-rank test, a statistical procedure specifically designed for survival analysis, was employed to rigorously examine the observed survival curves. The evaluation focused on determining the statistical significance of differences in survival outcomes among distinct groups or conditions. To ensure a robust and reliable evaluation of the data, a predetermined significance threshold of *p* < 0.05 was utilized to determine statistically meaningful associations.

## Results

### Variant landscape of programmed cell death genes in LUAD patients

**Figure 1:**
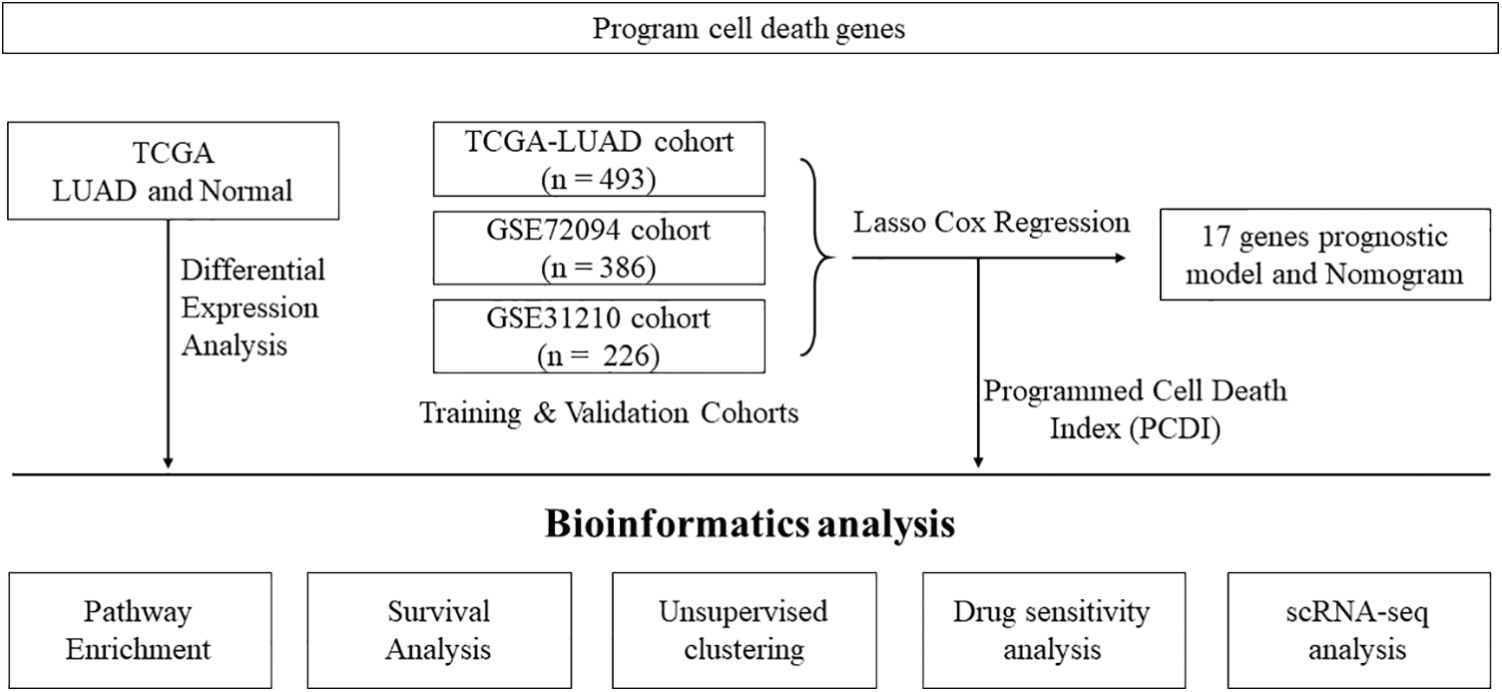
Flowchart illustrating the analysis workflow of this study.

**Figure 2:**
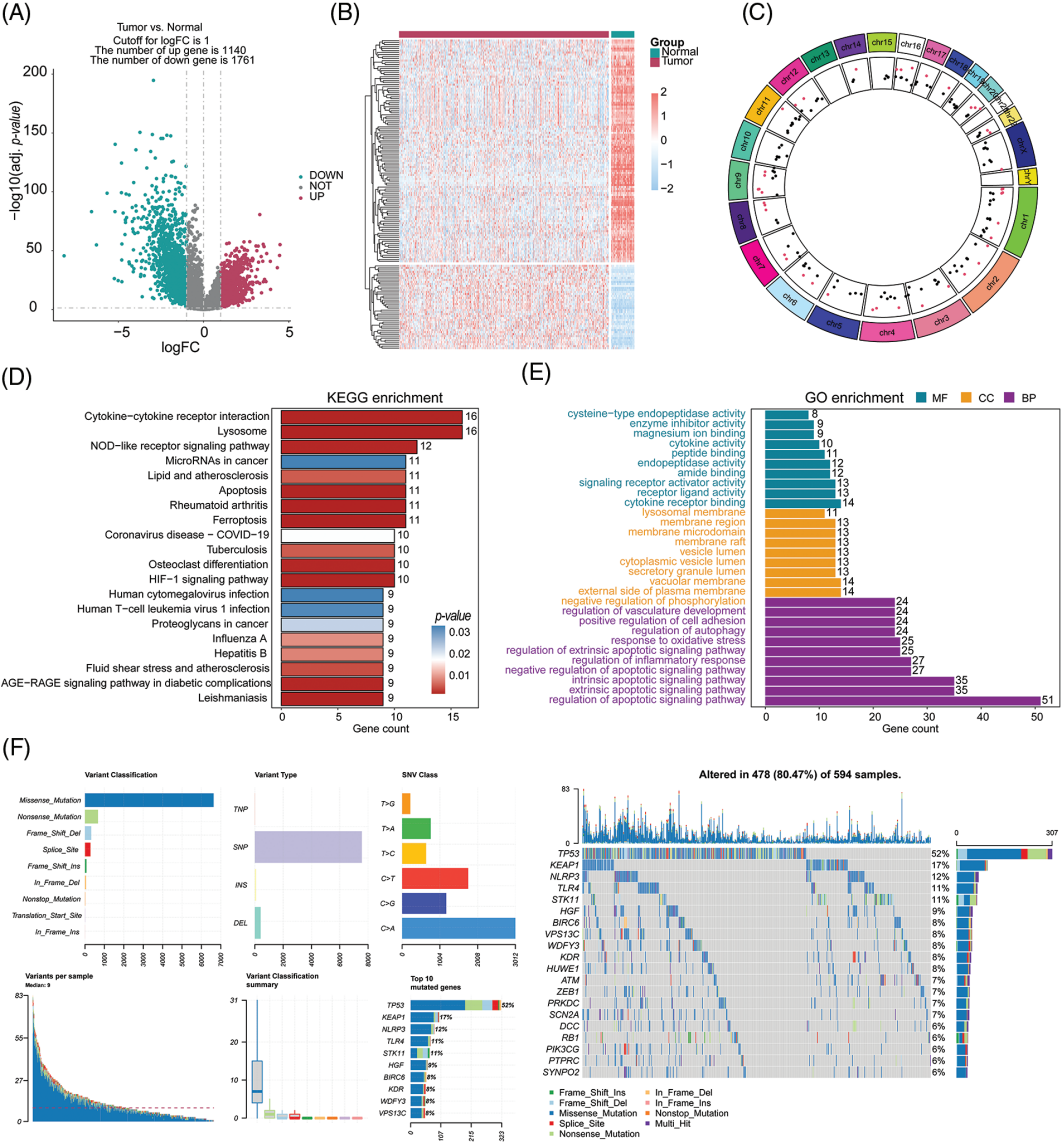
Selection and comprehensive analysis of differentially expressed genes. (A) Differential gene expression analysis between tumor samples (n = 539) and normal samples (n = 59). (B) Analysis of differential expression of 153 cell death-related genes in tumor samples and normal samples. (C) Circos plot showing the chromosomal locations of the 153 differentially expressed cell death-related genes. (D, E) KEGG and GO pathway enrichment analysis of the differentially expressed cell death-related genes. (F) Analysis of mutations in the top 20 cell death-related genes.

Please refer to Fig. 1 for the workflow of this study. In this study, we utilized the TCGA-LUAD cohort to identify 2,901 DEGs corrected *p*-values less than 0.05 and absolute log_2_FC greater than 1. In the cohort of LUAD samples, a total of 1,140 genes exhibited upregulation, while 1,761 genes demonstrated downregulation. The volcano plot in Fig. 2A displays the distribution of DEGs across the two groups, while the heatmap in Fig. 2B shows the proportion of differentially expressed RNA levels. Fig. 2C presents the chromosome positions, expression levels, and correlation of each DEG. Through KEGG and GO enrichment analyses, we discovered that these DEGs were involved in a broad range of biological pathways, including cytokine-cytokine receptor interaction, lysosome, extrinsic pathway of apoptosis, and regulation of programmed cell death signaling pathway (Figs. 2D and 2E). Furthermore, we performed an evaluation of the variation panorama of genes correlated with fatality in individuals diagnosed with LUAD, utilizing information from the TCGA group. Our analysis revealed that approximately 80.47% (478/594) of LUAD patients exhibited genetic mutations in these genes. Fig. 2F exhibits the foremost 20 mutations of genes associated with mortality, with TP53 demonstrating the highest mutation prevalence at 52%, while the other 19 mutations showed frequencies ranging between 6% and 17%. Our analysis of CNV status demonstrated frequent alterations of death-related genes.

### Prediction of optimal crosstalk genes and building the machine learning model

**Figure 3:**
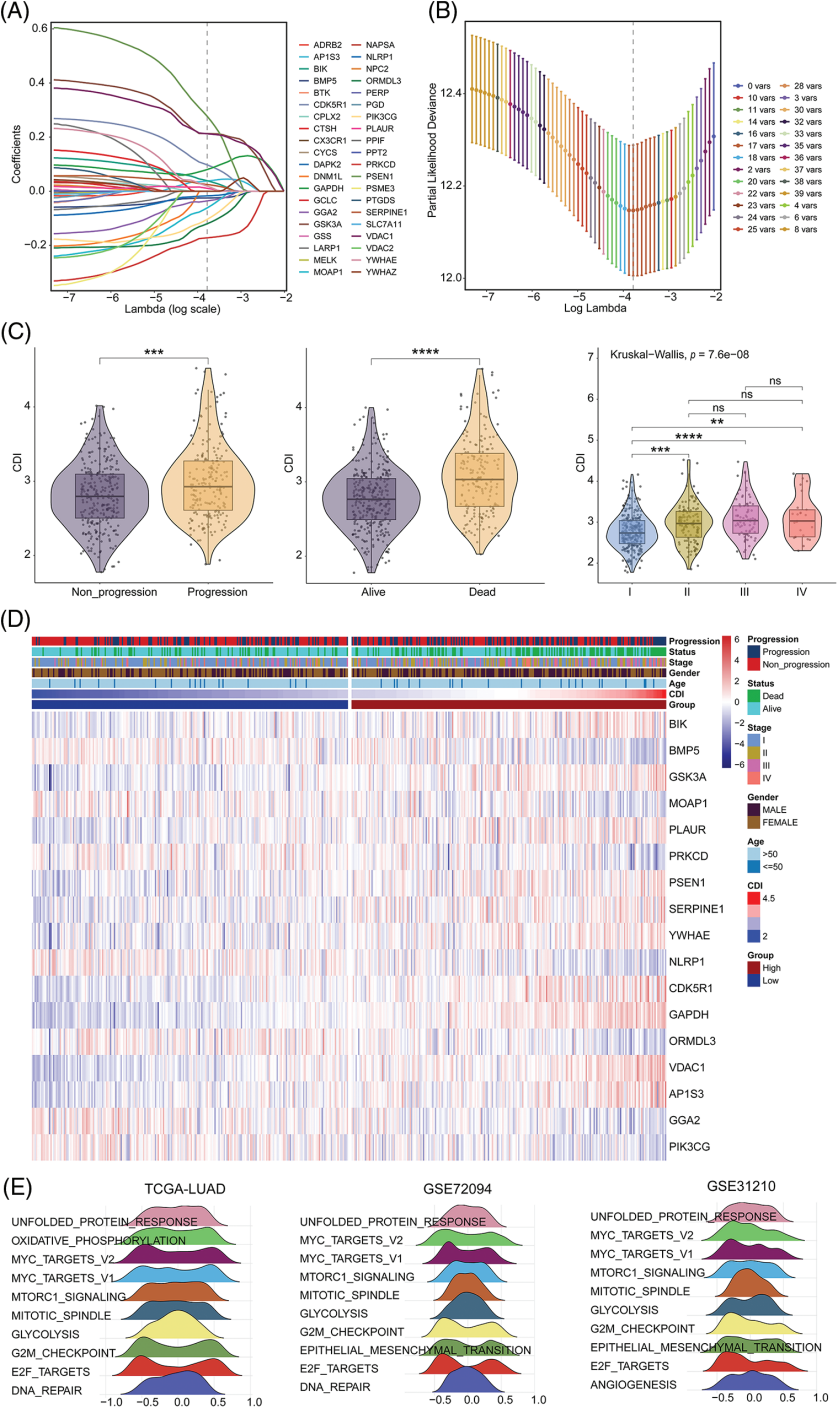
Model construction and comprehensive analysis. (A) Distribution plot of coefficients. (B) Cross-validation curve. (C) Correlation between cell death index (CDI) and clinical features. **p < 0.01; ***p < 0.001; ****p < 0.0001. (D) Heatmap showing the differences in model genes and clinical features between different CDI groups. (E) Identification of the top 10 pathways showing the most significant differences between different CDI groups in three datasets using GSVA analysis.

In this study, we employed a univariate Cox regression model to identify DEGs affiliated with apoptosis in both TCGA-LUAD and GSE72094 datasets, resulting in the identification of 40 prognostic death-related genes. Next, we applied the LASSO regression analysis using these genes to calculate correlation coefficients, which allowed for the identification of 17 optimal candidate genes (BIK, BMP5, GSK3A, MOAP1, PLAUR, PRKCD, PSEN1, SERPINE1, YWHAE, NLRP1, CDK5R1, GAPDH, ORMDL3, VDAC1, AP1S3, GGA2 and PIK3CG) for risk-scoring related to death prognosis (Figs. 3A and 3B) in the training set from TCGA-LUAD and GSE72094 datasets. The correlation and survival analysis among the CDI-related gene was shown in Suppl. Figs. S1 and S2. Furthermore, we conducted CDI and clinical pathological feature analysis, which revealed that pronounced expression of the 17 candidate genes was connected with severe disease escalation but low survival rates in LUAD, with significant differences observed among different stages (I, II, III, and IV) (Fig. 3C). It was also noted that the entirety of 17 CDI genes were significantly upregulated across clinical pathological features, as depicted in the heatmap (Fig. 3D). The different expression among the CDI-related genes was shown in Suppl. Fig. S3.

To better understand the biological processes underlying the identified gene signatures, we performed GSVA using the gene features to classify subgroups and identify disparities in biological processes. The heterogeneity in biological processes across subgroups was graphically illustrated employing a ridge plot, presenting the top ten prioritized biological processes within each cohort (Fig. 3E).

### Internal training and external validation of gene feature prediction model

**Figure 4:**
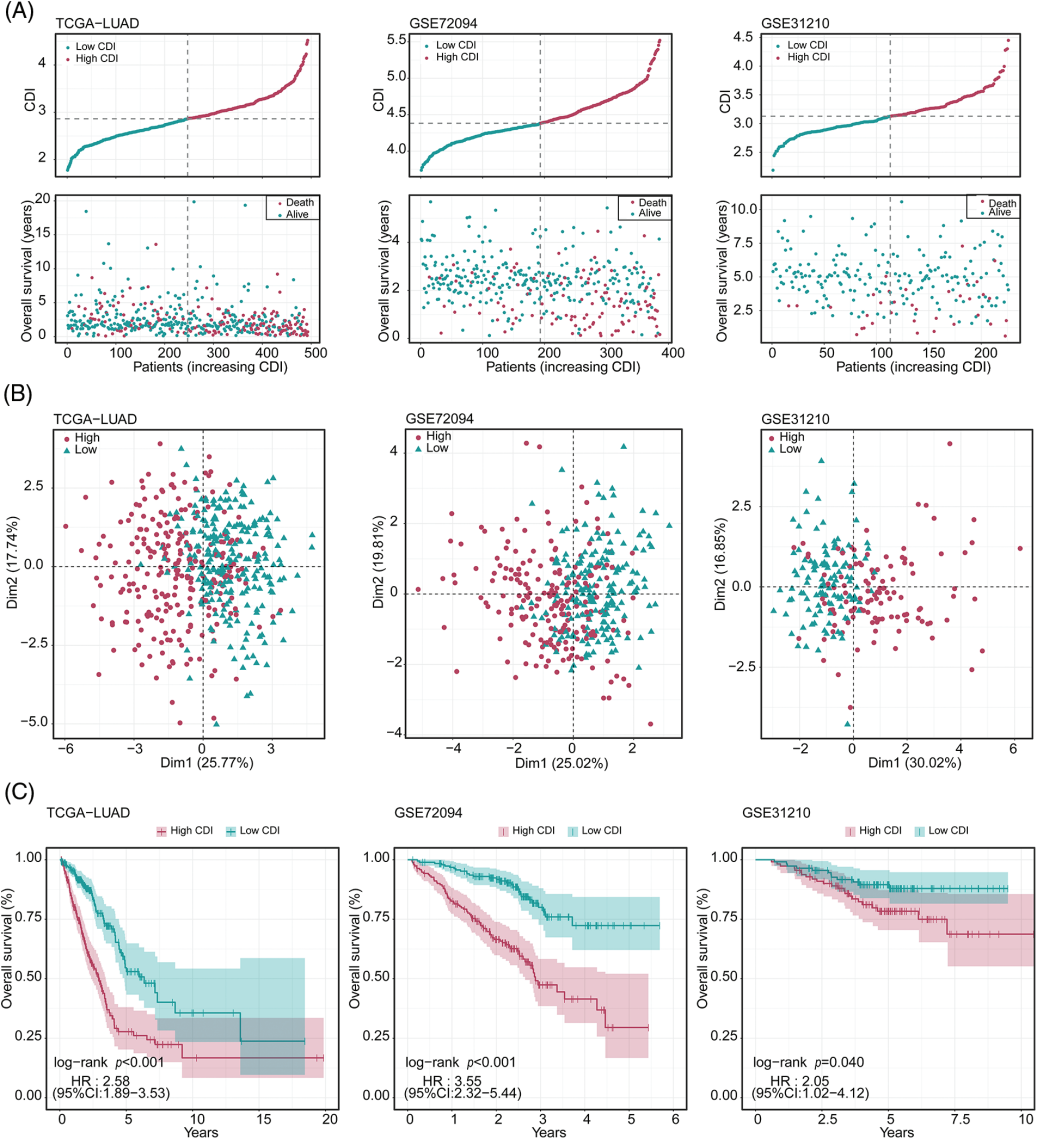
Evaluation of the predictive performance of cell death index (CDI) in three datasets. (A) Cumulative risk factor plot of three cohorts. (B) Principal component analysis (PCA) plot of three cohorts. (C) Kaplan-Meier survival curves of three cohorts.

To explore the prognostic significance of CDI in LUAD, we conducted a comparative analysis of the OS outcomes among patients exhibiting distinct CDI values. The outcomes of our investigation revealed a significant association between elevated CDI levels and diminished OS rates in the patient cohort under study, as demonstrated in Fig. 4A. Additionally, PCA was conducted to assess the categorization utilizing CDI. The findings demonstrated that the categorization utilizing CDI was deemed acceptable (Fig. 4B). We observed a significant discrepancy in the duration of survival overall between the low and high CDI cohorts, wherein individuals in the low CDI category exhibited markedly reduced mortality rates (*p* < 0.05, Fig. 4C).

To authenticate our discoveries, we used three independent cohorts: GSE72094, GSE31210, and KM-plotter. Using the median CDI values obtained from the validation cohorts, we partitioned 386 LUAD patients in GSE72094, 493 LUAD patients in TCGA, and 226 LUAD patients in GSE31210 into groups based on their CDI levels, specifically into high and low CDI categories. Our subsequent analysis of the data from all three cohorts confirmed our preliminary observations, indicating a significant association between high CDI and reduced overall lifespan (as illustrated in Fig. 4A). PCA analysis also confirmed the classification based on CDI (Fig. 4B). KM analysis unveiled that individuals belonging to the elevated CDI category exhibited reduced OS and elevated mortality rates across all three cohorts (all *p* < 0.05). On the other hand, patients classified within the low CDI group exhibited notably enhanced disease-free survival and freedom from recurrence were observed. Rates compared to their counterparts in the high CDI group (all *p* < 0.05, as depicted in Fig. 4C).

### Unsupervised clustering of programmed cell death related model genes

**Figure 5:**
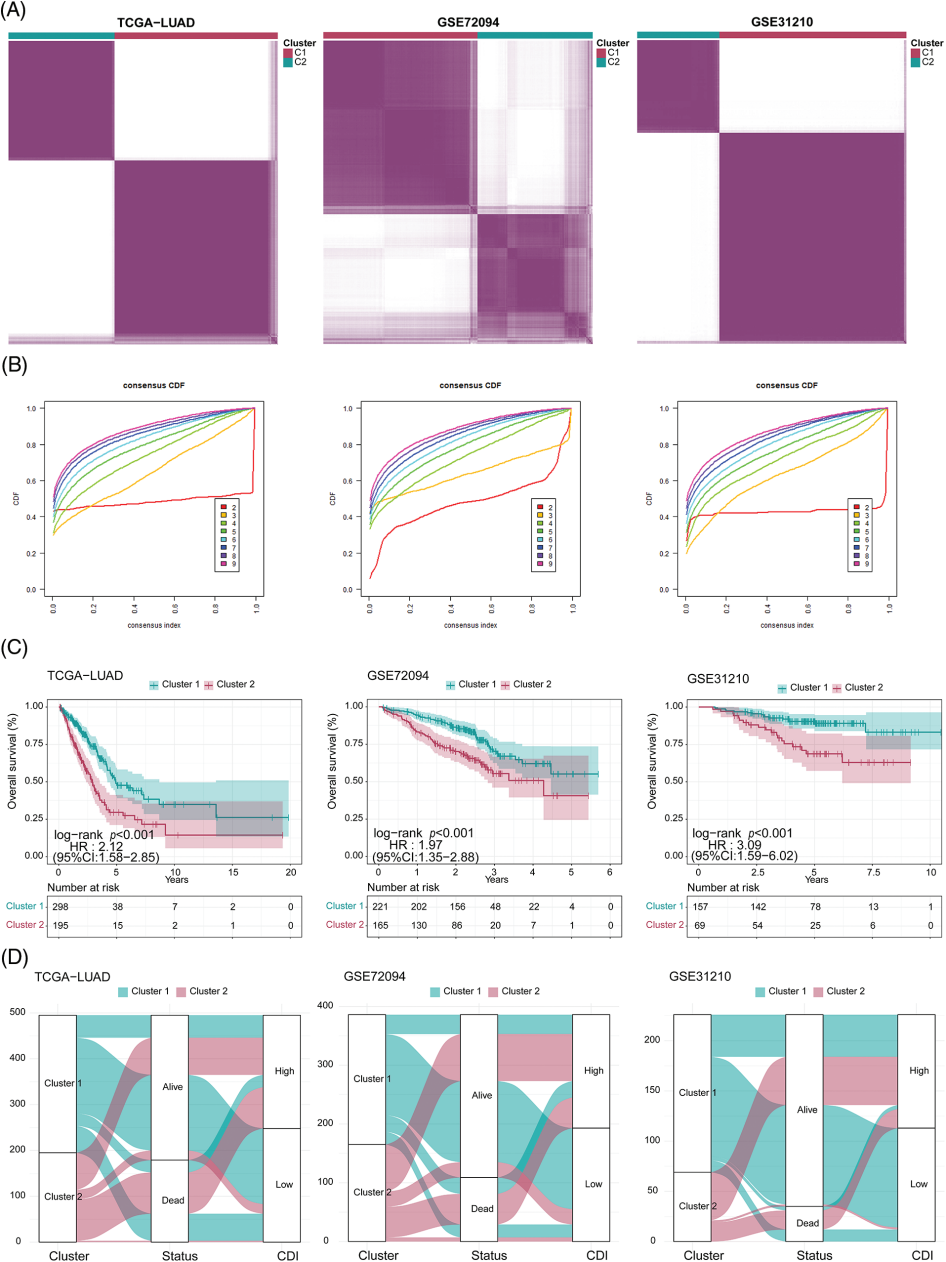
Clustering analysis for three cohorts. (A) Heatmap of the clustering matrix for three cohorts when k = 2. (B) Cumulative distribution function (CDF) curve of three cohorts. (C) Comparison of survival differences between clusters in three cohorts. (D) Sankey diagram (clusters, status, and CDI) of three cohorts.

To delineate potential subtypes within the context of LUAD, we employed an unsupervised clustering analysis incorporating 17 model genes associated with PCD. Strikingly, the dissimilarities between subgroups reached the pinnacle of statistical significance at k = 2, thus affirming the robust classification of 115 LUAD patients into two distinct clusters (Figs. 5A and 5B). Notably, these clusters displayed notable disparities in OS time (*p* < 0.001, Fig. 5C), thus highlighting the potential of PCD-associated genes as prognostic biomarkers for LUAD. Cluster 2 exhibited a favorable prognosis, while cluster 1 was characterized by a poor prognosis. Encouragingly, consistent findings were corroborated across additional cohorts, namely TCGA-LUAD (*p* < 0.001), GSE72094 (*p* < 0.001), and GSE31210 (*p* < 0.001) (Figs. 5A–5C).

As illustrated in Fig. 5D, the data revealed a clear correlation between the patients’ cluster membership and CDI severity, with a significant portion of the patients in cluster 1 showing reduced CDI while the majority of the patients in cluster 2 presenting with elevated CDI.

### Establishment and assessment of the nomogram survival model

**Figure 6:**
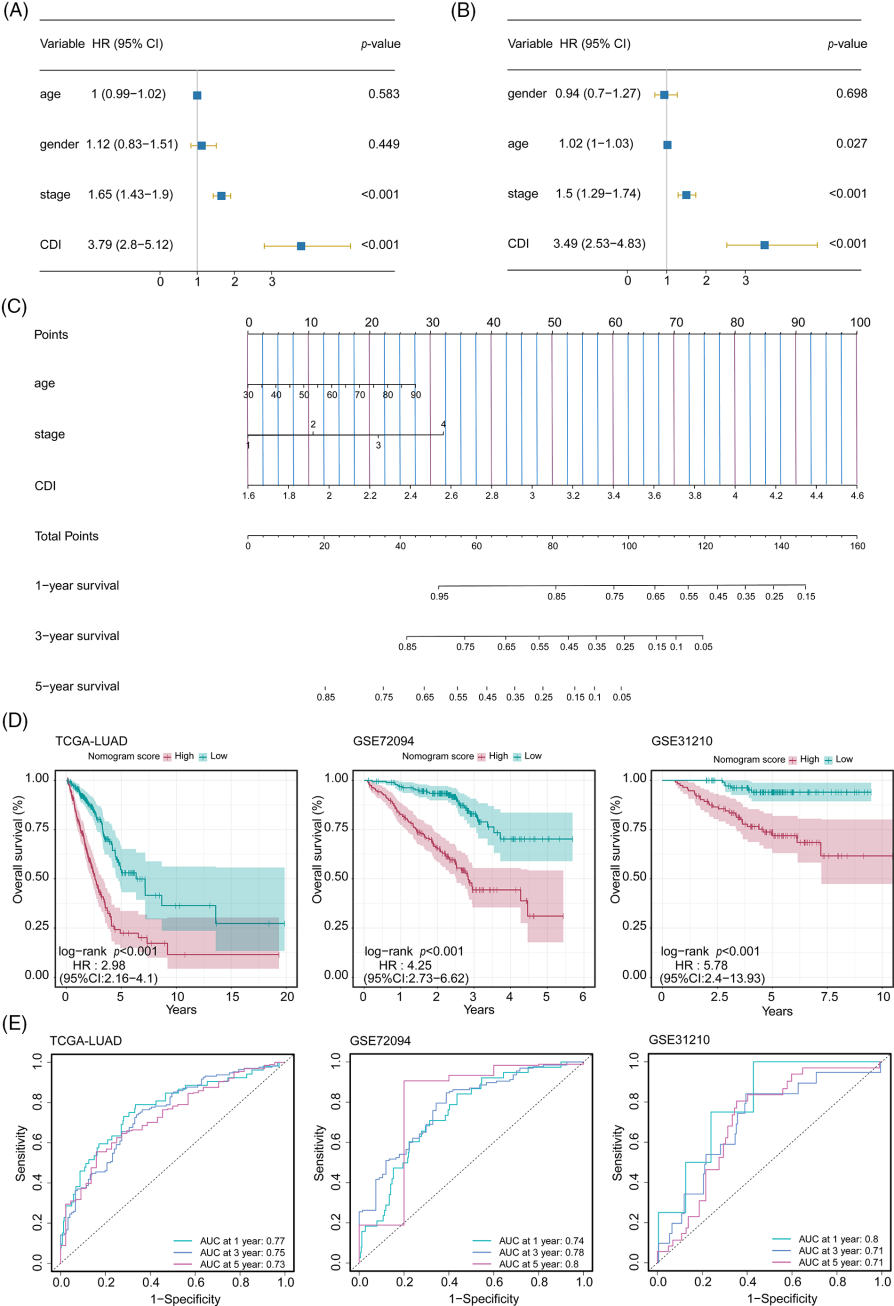
Development and performance evaluation of the nomogram. (A) Forest plot of univariate Cox regression analysis (including factors: age, gender, stage, and CDI). (B) Forest plot of multivariate Cox regression analysis (including factors: age, gender, stage, and CDI). (C) Nomogram model (including factors: age, stage, and CDI). (D) Survival curves of the nomogram model for three cohorts. (E) ROC curves of the nomogram model for three cohorts.

In order to assess the feasibility of CDI as an autonomous prognostic indicator, comprehensive univariate and multivariate Cox proportional hazards regression analyses were conducted. The univariate Cox proportional hazards regression analysis unveiled that compared to diverse components. The CDI constituted a significant danger (HR = 3.79, 95% CI: 2.8–5.21, and *p* < 0.001, as depicted in Fig. 6A). Further, after controlling for other covariates, the consequences of the multivariate analysis substantiated the significance of CDI as a self-reliant prognostic determinant in patients with LUAD (HR = 3.49, 95% CI: 2.53–4.83, *p* < 0.001, as shown in Fig. 6B). Subsequently, utilizing a comprehensive combination of multivariate Cox analysis and stepwise regression methodologies, we successfully constructed a robust nomogram model within the TCGA cohort, enabling estimation of 1-year, 3-year, and 5-year OS. Age, stage, and CDI were incorporated in the model (Fig. 6C). Calibration curve and Decision curve were shown in Suppl. Fig. S4. To assess the efficacy of the nomogram model, we assessed its calibration through a calibration curve, which illustrated the precision of the model in forecasting the survival rates at 1-year, 3-year, and 5-year intervals. (Suppl. Fig. S4A). Moreover, the application of DCA demonstrated the superior performance of the nomogram model in comparison to all other prognostic variables employed within this investigation (Suppl. Fig. S4B). Furthermore, an assessment of the area under the curve values was conducted across three distinct cohorts to ascertain the precision and reliability of the nomogram model in prognosticating the 1-year, 3-year, and 5-year survival rates among patients diagnosed with LUAD, as visually depicted in Fig. 6D.

### Dissection of Tumor Microenvironment and immune therapy response

**Figure 7:**
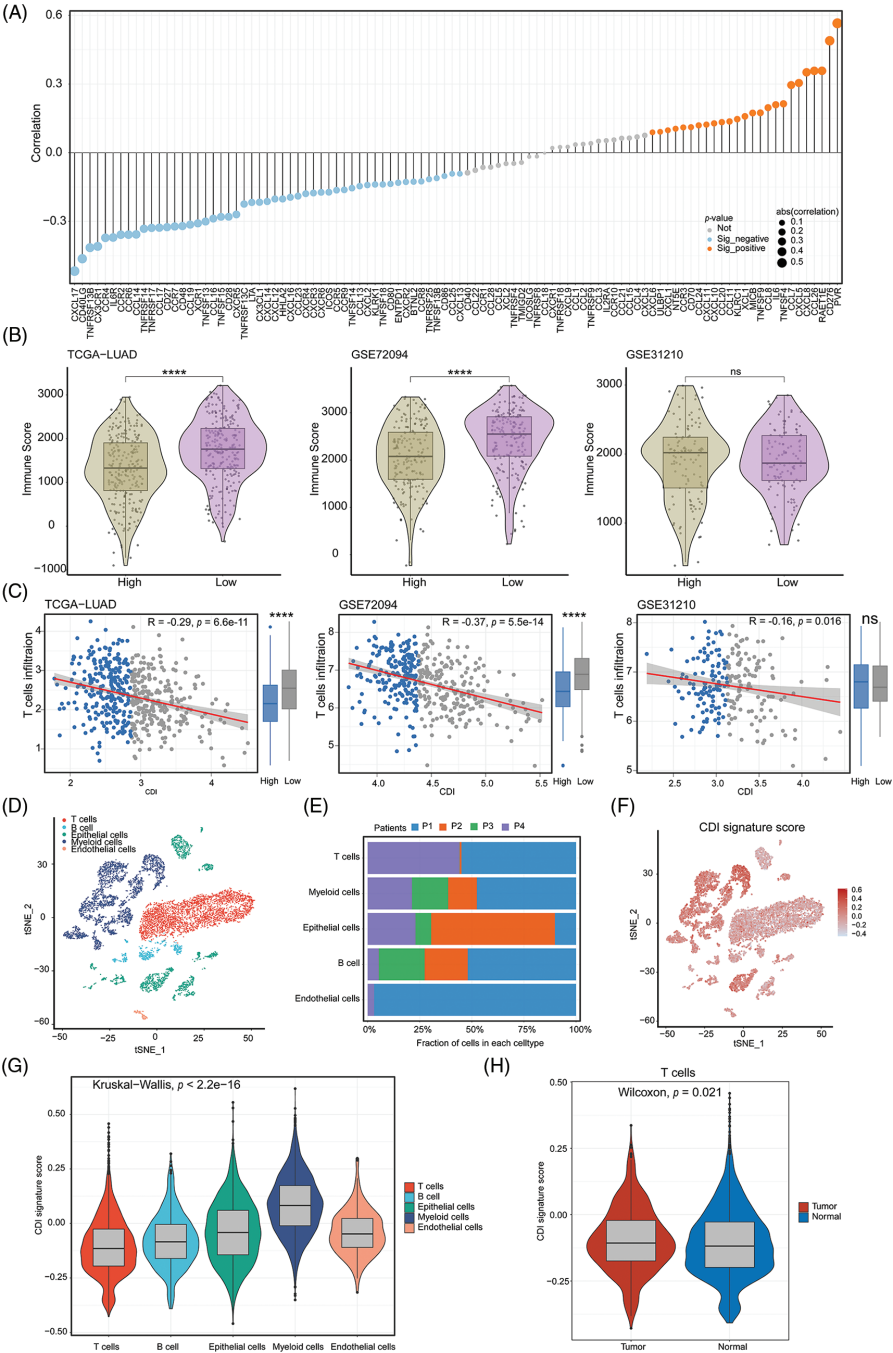
Immune-related analysis and single-cell analysis of CDI. (A) Correlation lollipop plot of CDI and immune-related genes. (B) Immune scores analysis in high CDI and low CDI groups from three cohorts using ESTIMATE algorithm. ****p < 0.0001. (C) Correlation analysis between CDI and T cell infiltration in three cohorts. (D) Annotation of cell subtypes using tSNE algorithm. (E) Proportions of cell subtypes in single-cell sequencing dataset of four patients. (F) Distribution of CDI values in cell subtypes. (G) Differential analysis of CDI among different cell subtypes. (H) Comparison of CDI difference between T cells in normal and tumor tissues.

To discern variances in additional pivotal attributes amidst the two distinct groups of CDI, we assessed the potential dissimilarity in the correlation between immune regulators and CDI values. The bar graph demonstrated a notable association between lower CDI values and heightened immune activity (Fig. 7A). Intriguingly, across all three cohorts, a notable rise in the immune score was detected in the low CDI cohort as compared with the high CDI group indicating a pronounced immune response in the former, and it was significantly negatively correlated with CDI (Figs. 7B and 7C). To delve deeper into the comprehensive distribution of CDI among LUAD patients, we employed single-cell RNA sequencing data (GSE72094 and GSE31210) to scrutinize the predominant cellular lineages (Figs. 7D and 7E). Our findings were consistent with the previous results, the data demonstrated an enrichment of CDI in tumor cells compared to other cellular subtypes (Figs. 7F and 7G). Additionally, compared with normal tissues, the CDI model score was more weakly expressed in T cells (*p* = 0.021, Fig. 7H). These results indicate that CDI index can be used as a basis for evaluating the heterogeneity of immune microenvironment and immune checkpoint inhibitor therapy.

### Programmed cell death signature in predicting drug sensitivity

**Figure 8:**
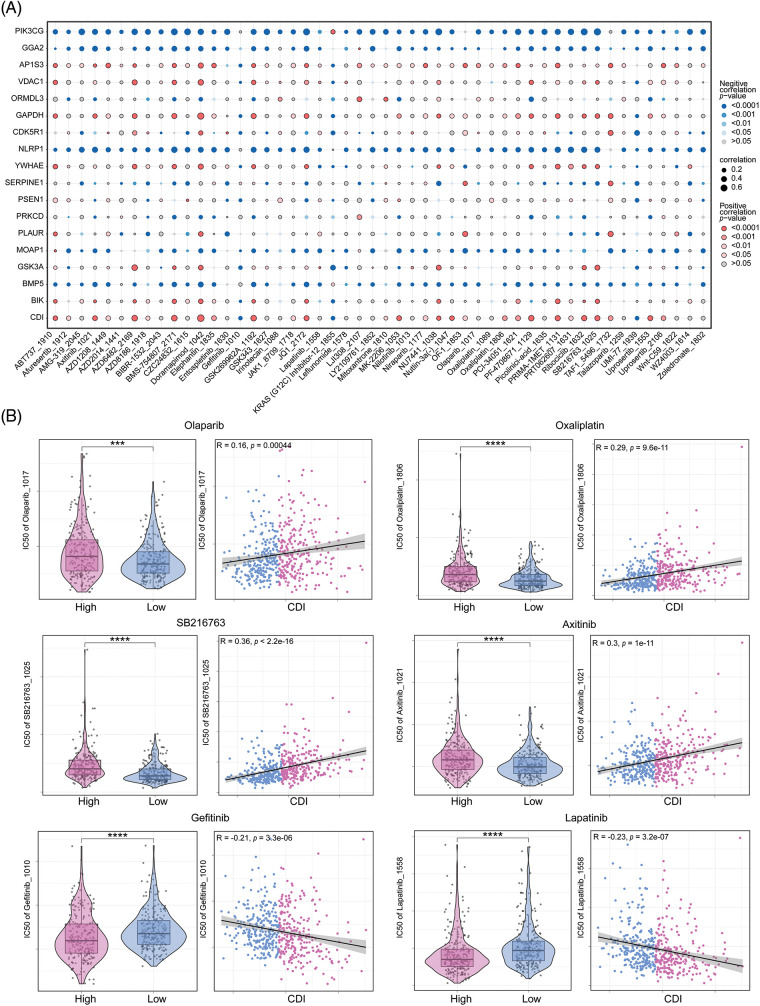
Drug sensitivity analysis. (A) Correlation dot plot between model genes and drugs. (B) Analysis of the correlation between six commonly used drugs and CDI, as well as the differential analysis of drug IC50 values between different CDI groups. ***p < 0.001; ****p < 0.0001.

With the intention of scrutinizing investigate the correlation between the PCD signature model and drug responsiveness, we performed an evaluation on the half-maximal inhibitory concentration values of various pharmacological compounds in specimens of LUAD. Observed were significant statistical differences in half-maximal inhibitory concentration (IC50) values among the cohorts stratified into high and low CDI status. Specifically, we noted that the IC50 values of Gefitinib and Lapatinib exhibited elevated levels within the high CDI cohort, while those of Olaparib, Oxaliplatin, SB216763, and Axitinib were lower in TCGA-LUAD dataset. The association and statistical importance between drug sensitivity and CDI are depicted in Figs. 8A and 8B. These results imply that individuals with elevated CDI levels may be susceptible to certain medical conditions may exhibit resistance to conventional treatment plans involving chemical medications but may respond better to FDA-approved alternative therapies designed for LUAD. In particular, Olaparib and Oxaliplatin may represent promising therapeutic options for chemo-resistant LUAD patients.

### PRKCD inhibit the proliferation and migration of lung adenocarcinoma cells

**Figure 9:**
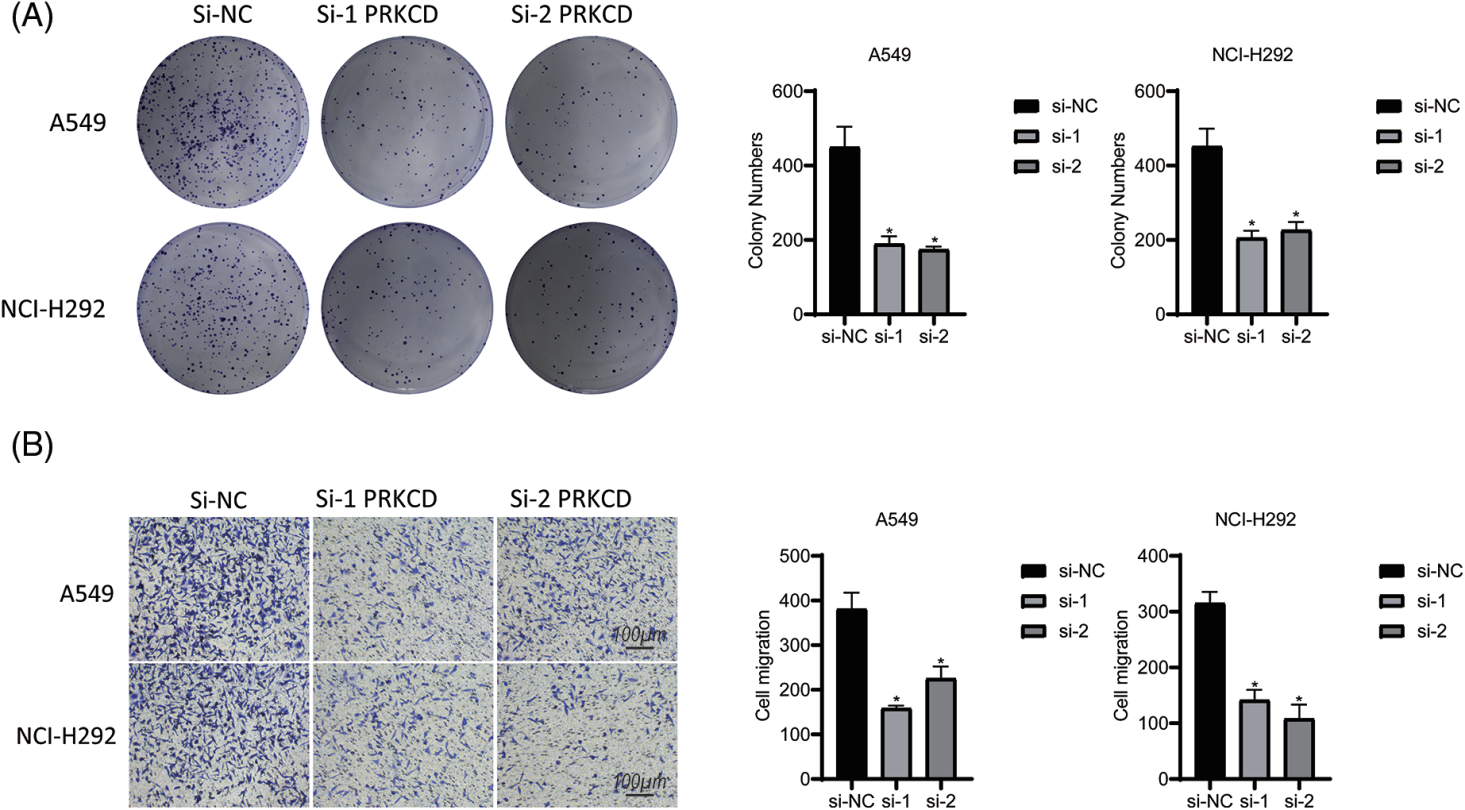
Cell assay in Lung carcinoma cell lines. (A) Colony formation assay in A549 and NCI-H292 cell lines: This experiment compared the cell colony-forming abilities among three groups: si-NC, si-1 PRKCD, and si-2 PRKCD. (B) Transwell assay in A549 and NCI-H292 cell lines: This experiment compared the cell migration abilities among three groups: si-NC, si-1 PRKCD, and si-2 PRKCD. *p < 0.05.

The results of this investigation indicate that the modulation of PRKCD expression may represent a promising treatment strategy for suppressing the tumorigenic potential and metastatic capacity of LUAD cells. PRKCD exhibited the most significant experimental results among all the constituent genes of CDI. Therefore, we focused our research on PRKCD. Results from the colony formation assay indicated a significant decrease in cell proliferation in both si1-PRKCD and si2-PRKCD groups, compared to the Si-NC group (Fig. 9A). Moreover, the results from the transwell assay demonstrated that the number of migrating cells significantly decreased in the si1-PRKCD and si2-PRKCD groups. This observation suggests a significant decline in cancer cell migration following the knockdown of PRKCD (Fig. 9B). These discoveries put forward that PRKCD could have a pivotal function in the onset and advancement of LUAD, and further investigation is warranted to elucidate its precise role in this disease.

## Discussion

The outcomes of this research provide valuable insights into the potential prognostic and therapeutic implications of the programmed cell death signature in LUAD. The results suggest that the CDI score may function as a dependable predictive indicator for LUAD, independent of other clinical factors. Furthermore, the study highlights the importance of the tumor microenvironment in influencing patient outcomes, as evidenced by the association between low CDI scores and higher immune activity. Finally, the investigation into drug sensitivity provides potential avenues for the development of targeted therapies in LUAD based on CDI signature status. Overall, the outcomes of this investigation carry significant ramifications for patient classification and tailored therapeutic strategies in LUAD.

Consistent with prior research, our discoveries highlight the significance of programmed cell death in both the progression and the curative outcomes of tumors. Previous investigations have exhibited that the expression of genes related to apoptosis serves as a promising biomarker for forecasting prognosis across various cancer types, such as breast cancer and gastric cancer [21,22]. Moreover, studies have evidenced that the programmed cell death pathway plays a crucial role in chemotherapy resistance in cancerous cells [23]. Our study expands upon these previous findings by presenting a distinct PCD signature that may serve as a prognostic indicator and possible predictor of drug sensitivity in LUAD.

One of the most intriguing findings of our research was the correlation between CDI score and immune activity. We found that higher immune activity was associated with low CDI scores. This finding aligns with previous studies that have shown that elevated immune activity is linked to better prognosis in many cancer types, including LUAD [24,25]. This suggests that programmed cell death may have a part to play in regulating the immune response within the tumor microenvironment. Additionally, our analysis of single-cell RNA sequencing data offered additional evidence that CDI is predominantly expressed in tumor cells, which may indicate a possible mechanism by which programmed cell death influences the immune response.

Our study also indicated that CDI score could potentially function as a predictor of drug sensitivity for certain FDA-approved drugs, such as Olaparib and Oxaliplatin. This aligns with prior research suggesting that PCD-related genes are associated with drug sensitivity in diverse cancer types, including ovarian cancer and colorectal cancer [26–28]. However, our study had its basis on *in silico* analysis, and additional experimental validation is essential to assess the clinical applicability of our findings.

Shize Pan et al. also constructed a PCD prognostic model for LUAD using bioinformatics techniques, which included 23 genes [29]. In comparison, our model has fewer genes (17 genes), making it more user-friendly. Additionally, we conducted wet experiments to validate the PRKCD gene within the model, yielding more convincing results. The protein encoded by PRKCD is a member of the serine/threonine-specific protein kinase protein kinase C family. It is highly expressed in many cancer cells and serves as both a tumor suppressor and a positive regulator of cell cycle progression. This protein can actively or negatively regulate cell apoptosis [30]. Our research suggests that PRKCD is an oncogene, and its high expression in lung cancer may promote cancer cell proliferation and migration. Based on existing studies and our results, we believe that the mechanisms involved with PRKCD are highly complex and warrant further investigation into its role in LUAD.

However, our study is subject to a number of constraints and constraints. Firstly, our analysis was conducted utilizing publicly available datasets, and as such, it warrants verification and validation with more extensive cohorts and diverse populations. Secondly, our research did not encompass functional experiments to elucidate the mechanisms underlying the link between the PCD signature and drug sensitivity. Further experimental inquiries are required to corroborate our conclusions. Thirdly, our study did not account for other essential factors, such as tumor mutation burden and neoantigen load, which have been demonstrated to be potential biomarkers for prognosticating and predicting drug sensitivity in LUAD [30,31].

Our scientific investigation has revealed that a prognostic model based on PCD signatures, as well as predictors of drug sensitivity in LUAD, can be established. Our analysis indicates that CDI score may be employed as a trustworthy biomarker for forecasting both result of surviving and drug sensitivity in LUAD. Additional research and experimentation are essential to fully ascertain the clinical usefulness of our findings.

## Supplementary Materials

Supplementary Figure 1 The correlation among cell death signature genes.

Supplementary Figure 2 The survival curve of cell death genes.

Supplementary Figure 3 The gene different expression in tumor and normal tissue.

Supplementary Figure 4 Assess the efficacy of the nomogram model.

## Data Availability

The datasets analyzed for this study can be found in the GEO website (https://www.ncbi.nlm.nih.gov/geo/).

## Supplementary Materials

The supplementary material is available online at https://doi.org/10.32604/or.2023.031134.
