# Supplementary figures and images for: Leveraging diverse cell-death patterns to predict the clinical outcome of immune checkpoint therapy in lung adenocarcinoma: Based on muti-omics analysis and vitro assay
Source: Oncol Res. 2023 Dec 28;32(2):393–407. doi: 10.32604/or.2023.031134 (PMC10765134; doi:10.32604/or.2023.031134)

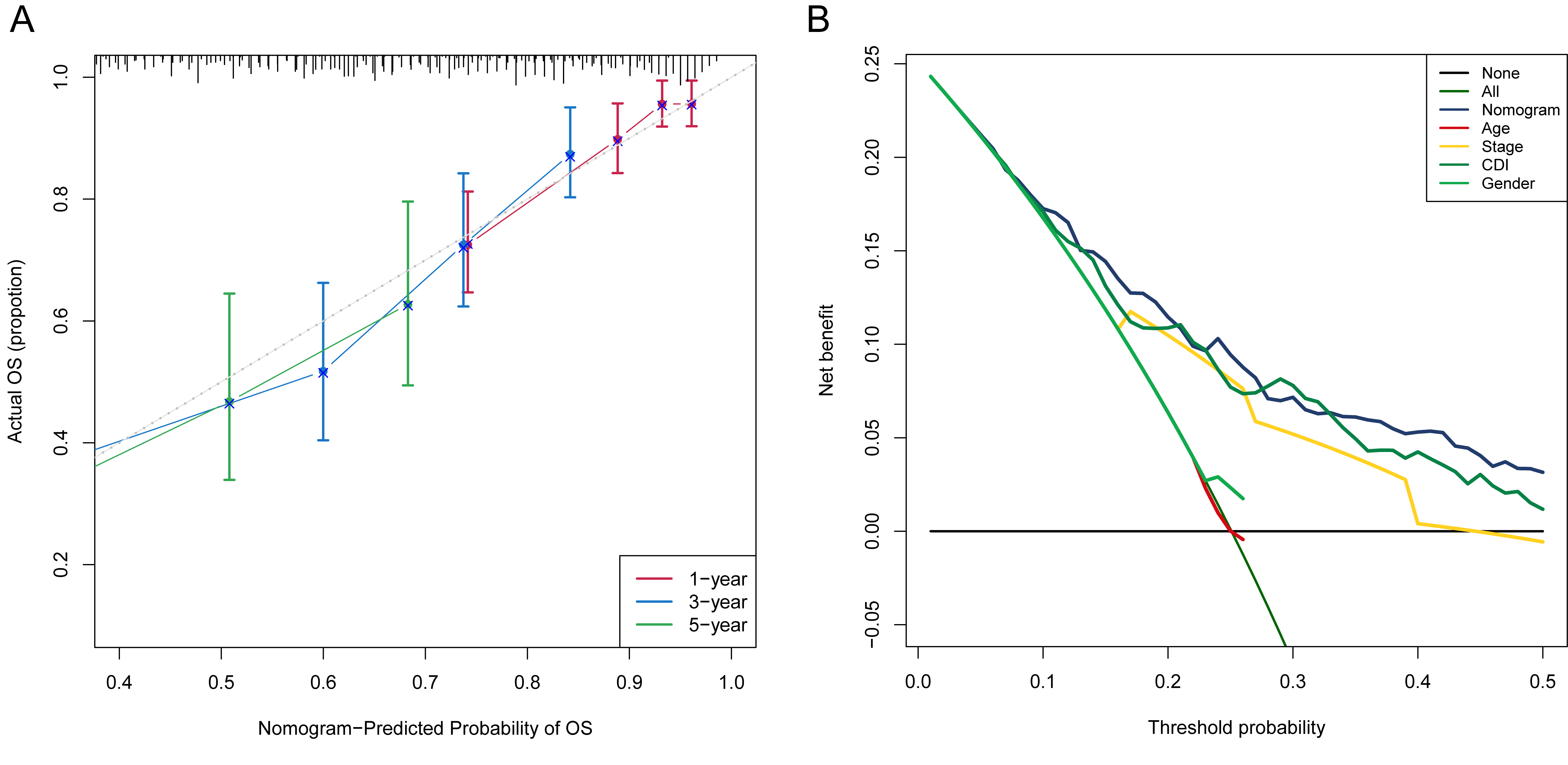

Supplement: Supplementary Figure 4 [file OncolRes-32-31134-s004.tif]
